# Exploring genetic association of systemic iron status and risk with incidence of diabetic neuropathy

**DOI:** 10.1186/s13098-024-01418-5

**Published:** 2024-07-25

**Authors:** Xinyue Yu, Tianyu Jin, Luyi Zhu, Shunyuan Guo, Binbin Deng, Yifan Cheng

**Affiliations:** 1 Alberta Institute, Wenzhou Medical University, Wenzhou, China; 2 Center for Rehabilitation Medicine, Department of Neurology, Zhejiang Provincial People’s Hospital (Affiliated People’s Hospital), Hangzhou Medical College, Hangzhou, Zhejiang China; 3 Department of Rehabilitation Medicine, the Second Affiliated Hospital, Yuying Children’s Hospital of Wenzhou Medical University, Wenzhou, China; 4 Department of Neurology, First Affiliated Hospital of Wenzhou Medical University, Wenzhou, China

**Keywords:** Diabetic neuropathy, Iron status, Causality, Mendelian randomization

## Abstract

**Background:**

Diabetic neuropathy (DN), a frequent complication in individuals with diabetes mellitus (DM), is hypothesized to have a correlation with systemic iron status, though the nature of this relationship remains unclear. This study employs two-sample Mendelian randomization (MR) analysis to explore this potential genetic association.

**Methods:**

We used genetic instruments significant associated with iron status including serum iron, ferritin, transferrin, and transferrin saturation, derived from an extensive Genome-Wide Association Study (GWAS) undertaken by the Genetics of Iron Status Consortium, involving a cohort of 48,972 European ancestry individuals. Summary statistics for DN were collected from a public GWAS, including 1,415 patients and 162,201 controls of European descent. Our MR analysis used the inverse-variance-weighted (IVW) method, supplemented by MR-Egger, weighted-median (WM) methods, Cochran’s Q test, MR-Egger intercept analysis, MR-Pleiotropy Residual Sum and Outlier (MR-PRESSO) method, and leave-one-out analysis to ensure robustness and consistency of the findings.

**Results:**

No genetic causal relationship was found between iron status markers and DN (all IVW *p* value > 0.05). Interestingly, a causative effect of DN on ferritin (IVW: OR = 0.943, 95% CI = 0.892–0.996, *p* = 0.035) and transferrin saturation (IVW: OR = 0.941, 95% CI = 0.888–0.998, *p* = 0.044) emerged. Sensitivity analyses confirmed the absence of significant heterogeneity and horizontal pleiotropy.

**Conclusion:**

While systemic iron status was not found to be causally related to DN, our findings suggest that DN may increase the risk of iron deficiency. These results provide further evidence supporting iron supplementation in patients with DN.

**Supplementary Information:**

The online version contains supplementary material available at 10.1186/s13098-024-01418-5.

## Introduction

Diabetic neuropathy (DN) is a frequent companion of diabetes mellitus (DM), affecting nearly half of diabetes patients [1]. With global diabetes rates projected to reach 693 million adults by 2045, DN occurrence is set to escalate correspondingly [2]. DN manifests as a progressive degeneration of peripheral nerves, especially sensory, motor, and autonomic fibers, resulting in pain, sensory disturbances, severe disability, and foot ulcers [1, 3]. These complications significantly impair patients’ quality of life and place considerable economic burdens on families and healthcare systems [2]. Despite extensive research, the exact pathophysiology of DN remains elusive. Chronic inflammation and oxidative stress from hyperglycemia, lipid metabolism dysfunctions, and irregular insulin signaling pathways are major contributors [4–6]. Recent studies suggest iron status may also influence DN progression.

Iron is crucial to various physiological functions including erythropoiesis, cellular metabolism, redox balance, and inflammation [7]. Both iron deficiency and excess can have harmful effects. Iron overload is known to intensify oxidative stress and inflammation, contributors to DN [8, 9]. Experimental models indicate iron deficiency, rather than overload, worsens neuropathy [10, 11]. Human epidemiological studies have linked iron status and dietary iron intake to hyperglycemia risk [12–15].

Mendelian randomization (MR) employed genetic variations as instrumental variables (IVs) to determine causality between modifiable exposures and health outcomes [16]. MR anaylsis offers the advantage of simulating randomized controlled trials as it relies on the random distribution of inherited genetic variants. Consequently, it can avoid typical observational study limitations such as confounding factors and reverse causality [17]. Furthermore, MR anaylsis is also cost-effective, providing an economical method for unbiased causal effect estimation, unlike resource-intensive randomized controlled trials [18]. To further explore the role of systemic iron status in DN, we used an MR analysis to investigate the causal relationship between systemic iron status biomarkers and DN risk.

## Methods

### Ethics statement

This MR study employed only publicly available, published Genome-Wide Association Study (GWAS) data. Ethical approval and informed consent were obtained for each participant as documented in the original publications and associated consortiums.

### Patient and public involvement statement

For FinnGen, the study protocol (Nr HUS/990/2017) was approved by The Coordinating Ethics Committee of the Hospital District of Helsinki and Uusimaa (HUS). For the Genetics of Iron Status Consortium, detailed ethical affirmations for each cohort are accessible in Supplementary Material 1 at 10.1038/ncomms5926.

### Study design and data source

**Fig. 1:**
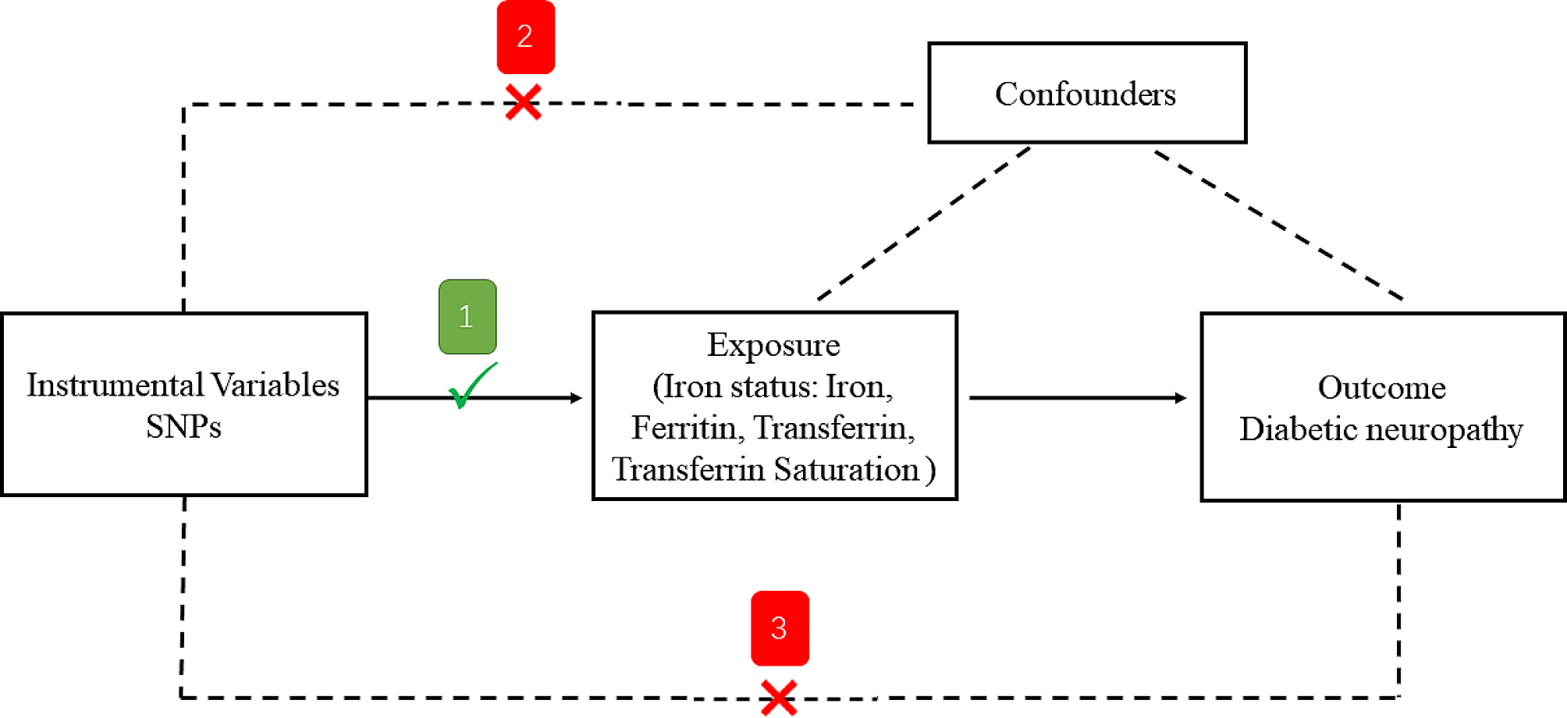
An overview of the study design. SNP: single nucleotide polymorphisms

In our two-sample MR study, which explored the potential causal relationship between iron status and DN risk, we identified single nucleotide polymorphisms (SNPs) as IVs for iron status. These SNPs met three criteria: (1) strong correlation with the iron status exposure; (2) no association with potential confounders; and (3) impact on the outcome (DN) exclusively through the exposure (iron status) (Fig. 1).

Four iron status biomarkers-iron, ferritin, transferrin, and transferrin saturation (TS), were selected as genetic instruments. We derived summary information of SNPs associated with iron status biomarkers from a prior meta-analysis of GWAS by the Genetics of Iron Status Consortium, involving 48,972 individuals of European descent from 19 cohorts [19]. The GWAS summary data for DN, comprising 1,415 instances and 162,201 control cases of European origin, came from the Finn consortium (https://www.finngen.fi/en). The characteristics and summarized data illustrating the association between SNPs and iron status biomarkers and DN are detailed in Table S1 and S2, respectively.

### Selection criteria for genetic variants

The SNPs extracted from GWAS that are associated with iron status are genome-wide significant (*p* < 5 × 10^− 8^) and independent (*r*^*2*^ ≤ 0.001) [20]. Then, PhenoScanner V2 (www.phenoscanner.medschl.cam.ac.uk) was used to eliminate SNPs associated with confounding factors of DN [21]. To preclude bias from weak instrumental variables, we ensured the F-statistics for each included SNP exceeded 10 [22]. The F-statistic formula is as follows, R^2^ = 2 × EAF × (1 − EAF) × β^2^, F statistic = R^2^ × (*N* − 2) / (1 − R^2^), where R^2^ represents the variance of exposed variability explained by individual IVs and N refers to the GWAS sample size of the exposure.

### Statistical analysis

We applied two-sample MR to investigate the causal link between iron status and DN. The inverse-variance-weighted (IVW), MR-Egger regression, and weighted median (WM) methods were used to estimate the effect value between iron status and DN. Our primary method hinged on the IVW approach, which consolidates the Wald ratios of individual SNPs through a meta-analytical process. The IVW method offers a stable estimate of the correlation between serum iron status and DN risk, assuming each genetic variant satisfies the IV assumptions [23].

Complementary to the IVW analysis, the MR-Egger regression and WM methods were further deployed to provide more robust results [24].

**Fig. 2:**
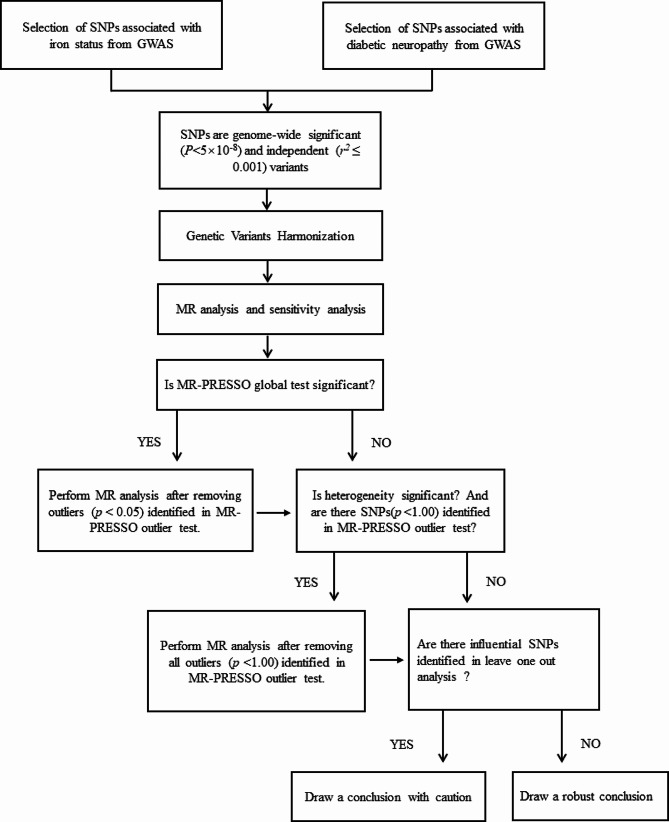
Workflow of Mendelian randomization study revealing causality from iron status on diabetic neuropathy. IVW: inverse variance weighted; MR: Mendelian randomization; MR-PRESSO: MR Pleiotropy RESidual Sum and Outlier; SNP: single-nucleotide polymorphisms

We also performed sensitivity analyses to assess potential heterogeneity and horizontal pleiotropy. To evaluate the heterogeneity of each genetic variant, we employed Cochran’s Q test (Cochran Q-derived *p* < 0.05) [25]. The MR-Egger regression method was used to explore the directional pleiotropy of the MR study [26] (we considered *p* < 0.05 as evidence of directional pleiotropy). MR-Pleiotropy Residual Sum and Outlier method (MR-PRESSO) was also performed to detect the potential horizontal pleiotropy and outliers [27] (global *P* < 0.05 implies the presence of horizontal pleiotropy). If significant horizontal pleiotropy was identified, we would remove the outlier variants to yield more precise corrected outcomes. Lastly, to ensure our results weren’t disproportionately impacted by individual SNPs, we executed a leave-one-out sensitivity analysis [28]. The flow chart of this study is shown in Fig. 2.

## Results

### Genetic instruments for iron status biomarkers

We finally extracted 16 significant and independent SNPs as genetic IVs, including three for iron, four for ferritin, five for transferrin and four for TS. The elevated F-statistics in our analysis, consistently exceeding the threshold of 10, confirm the minimal risk of weak IV bias. Detailed information of 16 SNPs is provided in Table S1. The summary statistics of SNPs for iron status and DN can be found in Table 1.

**Table 1:** SNPs from GWAS on systemic iron status and diabetic neuropathy

SNP	EA	OA	Exposure (Iron status)			Outcome (diabetic neuropathy)			
			β	SE	*p* value		β	SE	*p* value
**Iron**									
rs1525892	A	G	0.074	0.010	1.65E-12		0.062	0.042	0.142
rs1800562	A	G	0.372	0.020	3.96E-77		-0.123	0.102	0.229
rs855791	G	A	0.187	0.010	4.31E-77		0.010	0.041	0.808
**Ferritin**									
rs12693541	T	C	-0.106	0.014	4.18E-14		0.077	0.062	0.215
rs1800562	A	G	0.211	0.019	1.42E-29		-0.123	0.102	0.229
rs368243	C	T	-0.051	0.009	3.80E-08		0.038	0.039	0.329
rs2413450	C	T	0.056	0.010	3.57E-09		-0.002	0.040	0.966
**Transferrin**									
rs744653	T	C	0.092	0.014	2.00E-10		0.092	0.055	0.720
rs9990333	T	C	-0.067	0.010	3.01E-11		-0.067	0.040	0.266
rs17376530	T	C	-0.188	0.017	5.43E-30		-0.188	0.059	0.653
rs1800562	A	G	-0.550	0.021	1.26E-153		-0.550	0.102	0.229
rs174577	A	C	0.068	0.011	1.90E-10		0.068	0.040	0.025
**Transferrin saturation**									
rs8177272	A	G	-0.097	0.011	5.52E-20		0.062	0.042	0.142
rs1800562	A	G	0.577	0.020	1.52E-178		-0.123	0.102	0.229
rs221834	G	C	0.123	0.021	2.38E-09		0.021	0.082	0.796
rs855791	G	A	0.192	0.010	3.50E-80		0.010	0.041	0.808

SNP: single nucleotide polymorphisms; EA: effect allele; OA: other allele; SE, standard error. Iron status biomarkers data are publicly accessible via the Genetics of Iron Status Consortium at 10.1038/ncomms5926. Diabetic neuropathy data are openly available in FinnGen at https://www.finngen.fi/en

### Causal effect from iron status to DN

**Fig. 3:**
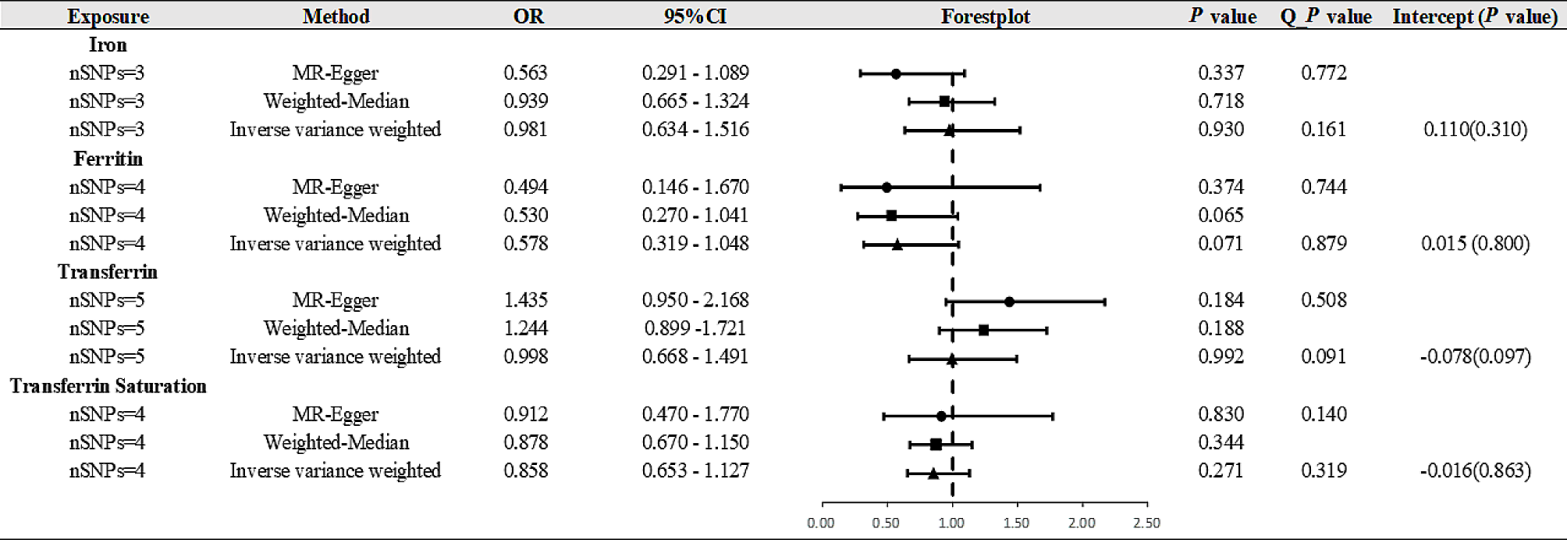
MR results and sensitivity analyses for association of iron status and diabetic neuropathy. OR: odds ratio; CI: confidence interval

The MR determinations obtained via various methodologies evaluating the causal impact of iron status on DN revealed a lack of causal relationships between the four examined iron biomarkers and DN (all *p* > 0.05). These findings are illustrated in Fig. 3.

**Fig. 4:**
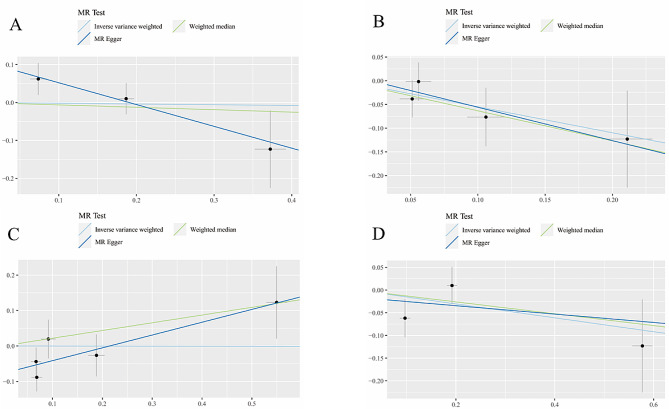
Scatter plots for MR analyses of the causal effect of iron status on diabetic neuropathy. **A** Iron, **B** Ferritin, **C** Transferrin, **D** Transferrin saturation

Our results show no causal relationship between serum iron (OR = 0.981, 95% CI: 0.634–1.516, *p* = 0.930), ferritin (OR = 0.578, 95% CI: 0.319–1.048, *p* = 0.071), transferrin (OR = 0.998, 95% CI: 0.668–1.491, *p* = 0.992), TS (OR = 0.858, 95% CI: 0.653 − 0.127, *p* = 0.271) with DN. Consistency in these findings was also seen in the MR-Egger (iron: OR = 0.563, 95% CI: 0.291–1.089, *p* = 0.337; ferritin: OR = 0.494, 95% CI: 0.146–1.670, *p* = 0.374; transferrin: OR = 1.435, 95% CI: 0.950–2.168, *p* = 0.184; TS: OR = 0.912, 95% CI: 0.470–1.770, *p* = 0.830) and WM methods (iron: OR = 0.939, 95% CI: 0.665–1.324, *p* = 0.718; ferritin: OR = 0.530, 95%CI: 0.270–1.041, *p* = 0.065; transferrin: OR = 1.244, 95% CI: 0.899–1.721, *p* = 0.188; TS: OR = 0.878, 95% CI: 0.670–1.150, *p* = 0.344). The relationships between individual iron status biomarkers and DN are graphically represented in Fig. 4, while the specific causal estimates from each of the 16 SNPs are displayed in Fig. S1.

To obtain reliable results, we performed sensitivity analysis to evaluate potential heterogeneity and horizontal pleiotropy, including Cochran’s Q test, the MR-Egger regression method, MR-PRESSO and leave-one-out analysis. The Cochran’s Q test for four iron biomarkers showed no significant heterogeneity (*p* = 0.161, 0.879, 0.091, and 0.319 for iron, ferritin, transferrin, and TS, respectively). Similarly, no significant evidence of horizontal pleiotropy was detected in this section (*p* for intercept = 0.310, 0.800, 0.097, and 0.863 for iron, ferritin, transferrin, and TS, respectively). The symmetry of the funnel plot indicated no evidence of pleiotropy (Fig. S2). Additionally, the leave-one-out analysis plots showed the absence of any highly influential SNPs affecting the causal relationship between iron status and DN (Fig. S3).

### Causal effect from DN to iron status

To further investigate the genetic causality linking DN and iron status, we conducted a reverse MR analysis, considering DN as the exposure and iron status as the outcome. Two SNPs that were significantly and independently associated with DN were identified (Table S3). The IVW method revealed a causal association of DN on ferritin and TS (ferritin OR = 0.943, 95% CI = 0.892–0.996, *p* = 0.035; TS OR = 0.941, 95% CI = 0.888–0.998, *p* = 0.044). However, there was no evidence of causality of DN on iron and transferrin (iron OR = 0.952, 95% CI = 0.898–1.010, *p* = 0.101; transferrin OR = 1.033, 95% CI = 0.972–1.097, *p* = 0.296). The Cochran Q-test did not exhibit significant heterogeneity for serum iron (*p* = 0.849), ferritin (*p* = 0.634), transferrin (*p* = 0.924) or TS (*p* = 0.878) (Table 2).

**Table 2:** Mendelian randomization estimates of the associations from diabetic neuropathy on iron status

Outcome	OR	95% CI	*p* value for IVW	*p* value for Cochran Q-test
Iron	0.952	0.898–1.010	0.101	0.849
Ferritin	0.943	0.892–0.996	0.035	0.634
Transferrin	1.033	0.972–1.097	0.296	0.924
Transferrin saturation	0.941	0.888–0.998	0.044	0.878

## Discussion

Utilizing a two-sample bidirectional MR analysis, we investigated the causal relationships between four iron status biomarkers and DN. Overall, our findings did not establish a causal connection between these biomarkers and the risk of DN; however, they indicated that DN might increase the risk of iron deficiency.

As an essential trace element, iron is critical for various metabolic processes in humans, including oxygen transport, energy metabolism, nucleotide synthesis, and electron transport [7]. Given the toxic nature of excessive iron, it’s vital to keep its concentration within a specific optimal range. Research has linked high iron storage to increased likelihood of conditions like DM [29, 30] and metabolic syndrome [31, 32]. Observational studies has demonstrated a correlation between high dietary iron intake and the risk of DN [33]. This association may stem from excess iron’s capacity to generate reactive oxygen species [8], and its role in promoting insulin resistance and diminishing insulin secretion by oxidizing lipids, proteins, and nucleic acids [9, 34].

In contrast, animal studies have yielded different conclusions. Petra Baum et al. revealed that a diet low in iron, rather than high, had a significant impact on the development of an STZ-induced experimental DN model [35]. This limited iron intake resulted in decreased sensory conduction velocities in the sciatic nerve and caused mitochondrial damage in dorsal root ganglion neurons [35]. Similar findings were observed in peripheral neuropathy model in obese *ob/ob* mice and T2DM *db/db* mice [10, 36]. A low dietary iron intake exacerbated inflammation and promoted peripheral nerve degeneration [37]. A high dietary iron intake reduced pro-inflammatory M1 macrophages in nerve sections and increased anti-inflammatory M2 macrophages [10].

Collectively, iron homeostasis is paramount in preserving the structure and integrity of the peripheral nervous system [38]. Few studies have focused on iron metabolism and peripheral neuropathy. Our study revealed two novel aspects. Firstly, we discovered no evidence that these four iron status biomarkers related to iron status were causally linked with DN risk, providing evidence that iron intake causes peripheral neuropathy not due to iron burden itself but possibly due to associated inflammatory activity within the peripheral nerves. Secondly, our finding implied that DN increased the likelihood of iron deficiency, which aligns with the observation that patients with DN are prone to combined anemia [39]. This provides further evidence for iron supplementation in patients with DN.

To our knowledge, this is the first MR study investigating the causal link between iron status and DN, using SNPs associated with four iron biomarkers from the largest meta-GWAS of European descent as IVs to derive estimates of DN risk impact. Our primary MR analysis employing the IVW method did not substantiate a causal effect of iron levels on susceptibility to DN, with consistent results affirmed by both MR-Egger and weighted median approaches. Additional assessments including Cochran’s Q test, MR-PRESSO, and leave-one-out analysis all indicated robust and reliable outcomes. In our reverse direction MR analysis that utilized DN as the exposure and iron status as the outcome, we found that DN was genetically associated with low serum transferrin concentration and low transferrin saturation level, suggesting that DN increases the risk of iron deficiency. Sensitivity analysis in the reverse direction MR analysis did not reveal significant heterogeneity. A significant strength of our study is the MR design, which mitigates confounding inherent in traditional observational studies by utilizing genetic variants as IVs.

While our MR analysis is robust, it has limitations. Its validity relies on three assumptions: the genetic variant is associated with the exposure, exerts its effect on the outcome solely through this exposure, and is not confounded by other factors. Violating these assumptions can lead to biased conclusions. Additionally, our study was restricted to European lineage, therefore, future research should investigate whether similar findings are observed in diverse ethnic populations. Furthermore, even though we utilized the most extensive and latest GWAS database, the scope of our study remains relatively modest when compared to the broad population-based observational studies. Lastly, the GWAS database lacked detailed demographics and clinical information, thus hindering further subgroup analyses.

## Conclusion

In conclusion, we employed two-sample MR analysis to explore the relationship between iron status and DN. These results did not indicate a causal association of iron status on the risk of DN. Nonetheless, our finding implied that DN increases the risk of iron deficiency, providing further evidence for iron supplementation in those with DN. Moving forward, advanced MR studies would be beneficial to verify our findings when more comprehensive GWAS summary data become available. Simultaneously, additional research needs to be pursed to discover predictive and prognostic markers for DN and to explore their possible pathogenic mechanisms. In addition, we also need to conduct further prospective cohort study to determine the recommended dietary intake of iron for those with DN.

## Electronic supplementary material

Below is the link to the electronic supplementary material.

Supplementary Material 1

Supplementary Material 2

## Data Availability

No datasets were generated or analysed during the current study.

## Abbreviations

DM: Diabetes mellitus;
DN: Diabetic neuropathy;
GWAS: Genome-Wide Association Study;
IV: Instrumental variable;
IVW: Inverse-variance-weighted;
LD: Linkage Disequilibrium;
MR: Mendelian randomization;
MR-PRESSO: MR-Pleiotropy Residual Sum and Outlier method;
SNPs: Single nucleotide polymorphisms;
STZ: Streptozotocin;
TS: Transferrin saturation;
WM: Weighted median
