# Correction: Exploring genetic association of systemic iron status and risk with incidence of diabetic neuropathy

**DOI:** 10.1186/s13098-024-01498-3

**Published:** 2024-10-30

**Authors:** Xinyue Yu, Tianyu Jin, Luyi Zhu, Shunyuan Guo, Binbin Deng, Yifan Cheng

**Affiliations:** 1 Alberta Institute, Wenzhou Medical University, Wenzhou, China; 2 Center for Rehabilitation Medicine, Department of Neurology, Zhejiang Provincial People’s Hospital (Affiliated People’s Hospital), Hangzhou Medical College, Hangzhou, Zhejiang China; 3 Department of Rehabilitation Medicine, the Second Affiliated Hospital, Yuying Children’s Hospital of Wenzhou Medical University, Wenzhou, China; 4 Department of Neurology, First Affiliated Hospital of Wenzhou Medical University, Wenzhou, China

**Correction: Diabetology & Metabolic Syndrome (2024) 16: 174**

10.1186/s13098-024-01418-5

In this article the first affiliation details for Yifan Cheng were incorrectly given as ‘Alberta Institute, Wenzhou Medical University, Wenzhou, China’ but should have been ‘Center for Rehabilitation Medicine, Department of Neurology, Zhejiang Provincial People’s Hospital (Affiliated People’s Hospital), Hangzhou Medical College, Hangzhou, Zhejiang, China”.

The original article has been corrected.

