# Supplementary material for: Exploring genetic association of systemic iron status and risk with incidence of diabetic neuropathy
Source: Diabetol Metab Syndr. 2024 Jul 25;16:174. doi: 10.1186/s13098-024-01418-5 (PMC11270780; doi:10.1186/s13098-024-01418-5)
Supplement: Supplementary file 1 — Supplementary Material 1 [file 13098_2024_1418_MOESM1_ESM.docx]

**Table S1 Characteristics of instrumental variables for Iron status**

| **SNP** | **EA** | **OA** | **Gene** | **EAF** | **β** | **SE** | ***p* value** | **Sample size** | **R^2^** | **F - statistic** |
| --- | --- | --- | --- | --- | --- | --- | --- | --- | --- | --- |
| **Iron** |  |  |  |  |  |  |  |  |  |  |
| rs1525892 | A | G | TF | 0.243 | 0.074 | 0.010 | 1.65E-12 | 48972 | 0.002 | 97.799 |
| rs1800562 | A | G | HFE | 0.067 | 0.372 | 0.020 | 3.96E-77 | 48972 | 0.017 | 864.036 |
| rs855791 | G | A | TMPRSS6 | 0.446 | 0.187 | 0.010 | 4.31E-77 | 48972 | 0.017 | 859.236 |
| **ferritin** |  |  |  |  |  |  |  |  |  |  |
| rs12693541 | T | C | SLC40A1 | 0.871 | -0.106 | 0.014 | 4.18E-14 | 48972 | 0.003 | 123.959 |
| rs1800562 | A | G | HFE | 0.068 | 0.211 | 0.019 | 1.42E-29 | 48972 | 0.006 | 277.912 |
| rs368243 | C | T | TEX14 | 0.440 | -0.051 | 0.009 | 3.80E-08 | 48972 | 0.001 | 63.344 |
| rs2413450 | C | T | TMPRSS6 | 0.463 | 0.056 | 0.010 | 3.57E-09 | 48972 | 0.002 | 76.210 |
| **transferrin** |  |  |  |  |  |  |  |  |  |  |
| rs744653 | T | C | WDR75, SLC40A1 | 0.854 | 0.092 | 0.014 | 2.00E-10 | 48972 | 0.002 | 102.677 |
| rs9990333 | T | C | NPFFR2, ADAMTS3 | 0.460 | -0.067 | 0.010 | 3.01E-11 | 48972 | 0.002 | 109.454 |
| rs17376530 | T | C | RAB6B | 0.106 | -0.188 | 0.017 | 5.43E-30 | 48972 | 0.007 | 329.631 |
| rs1800562 | A | G | HFE | 0.066 | -0.550 | 0.021 | 1.26E-153 | 48972 | 0.037 | 1894.203 |
| rs174577 | A | C | FADS2 | 0.333 | 0.068 | 0.011 | 1.90E-10 | 48972 | 0.002 | 101.987 |
| **Transferrin Saturation** |  |  |  |  |  |  |  |  |  |  |
| rs8177272 | A | G | TF | 0.331 | -0.097 | 0.011 | 5.52E-20 | 48972 | 0.004 | 204.914 |
| rs1800562 | A | G | HFE | 0.067 | 0.577 | 0.020 | 1.52E-178 | 48972 | 0.042 | 2128.365 |
| rs221834 | G | C | ZAN (TFR2, EPO) | 0.072 | 0.123 | 0.021 | 2.38E-09 | 48972 | 0.002 | 98.559 |
| rs855791 | G | A | TMPRSS6 | 0.554 | 0.192 | 0.010 | 3.50E-80 | 48972 | 0.018 | 909.604 |

SNP: single nucleotide polymorphisms; EA: effect allele; OA: other allele; EAF: effect allele frequency; SE, standard error

**Table S2 Characteristics of instrumental variables for Iron status**

| **SNP** | **EA** | **OA** | **Gene** | **EAF** | **β** | **SE** | ***p* value** | **Sample size** | **R^2^** | **F - statistic** |
| --- | --- | --- | --- | --- | --- | --- | --- | --- | --- | --- |
| rs635688 | C | T | BRD2 | 0.629 | 0.274 | 0.041 | 2.27E-11 | 163616 | 3.50E-02 | 5.93E+03 |
| rs6679677 | A | C | PHTF1 | 0.146 | 0.347 | 0.056 | 6.17E-10 | 163616 | 3.00E-02 | 5.06E+03 |

SNP: single nucleotide polymorphisms; EA: effect allele; OA: other allele; EAF: effect allele frequency; SE, standard error


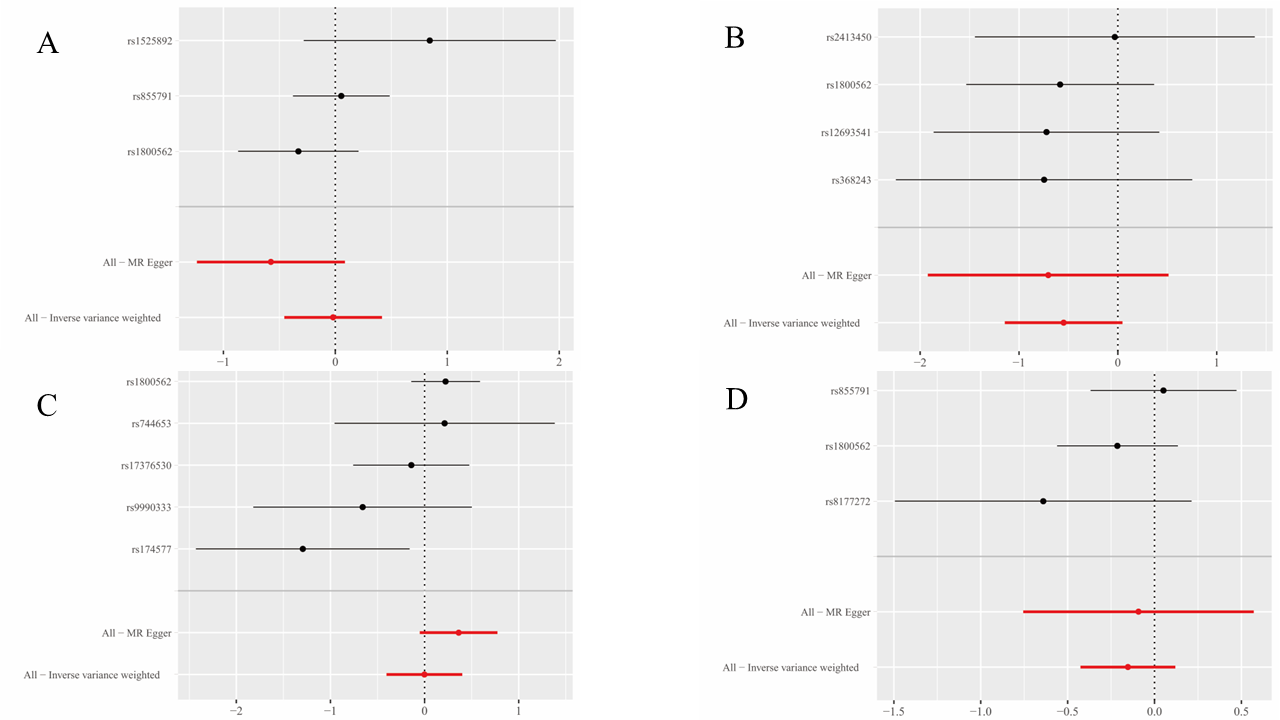


**Figure S1** Forest plot of ferritin (A), iron (B), transferrin (C), transferrin saturation (D) and diabetic neuropathy. The forest plot shows the individual causal estimates of each of the 16 SNPs.


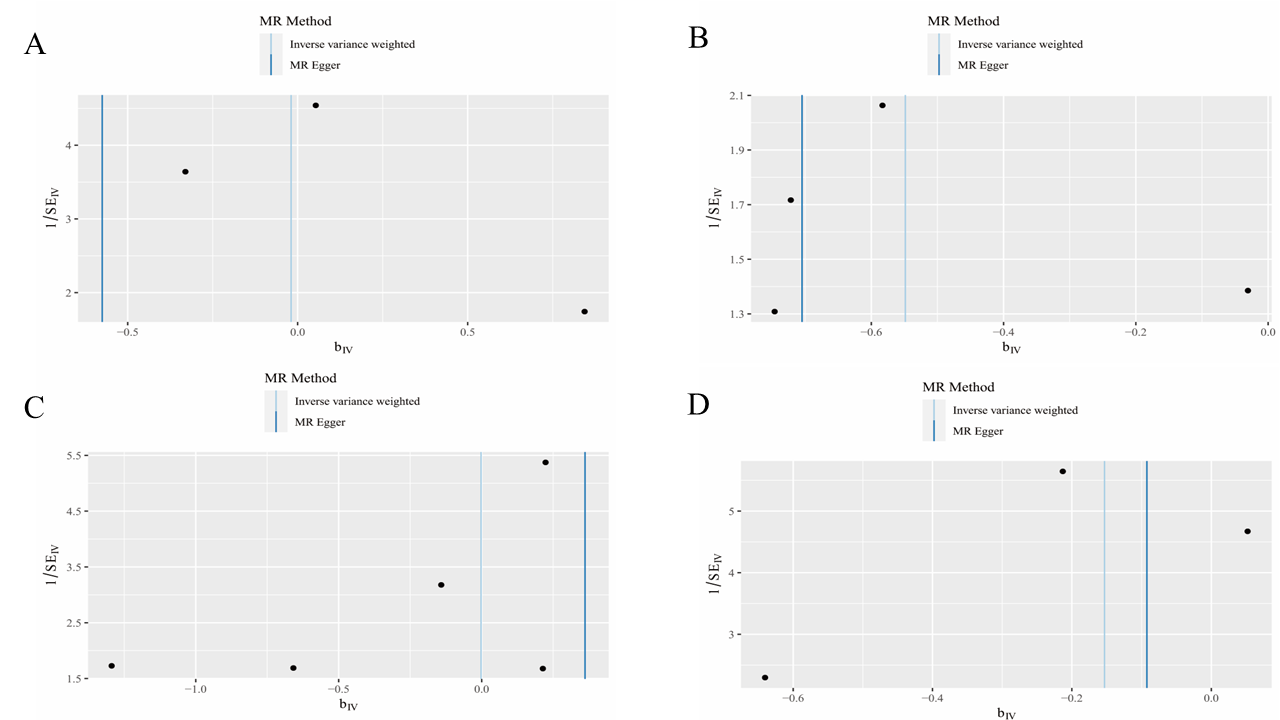


**Figure S2** Funnel plot of ferritin (A), iron (B), transferrin (C), transferrin saturation (D) and diabetic neuropathy. The funnel plots are symmetric, which indicates that the absence of polymorphism.

**
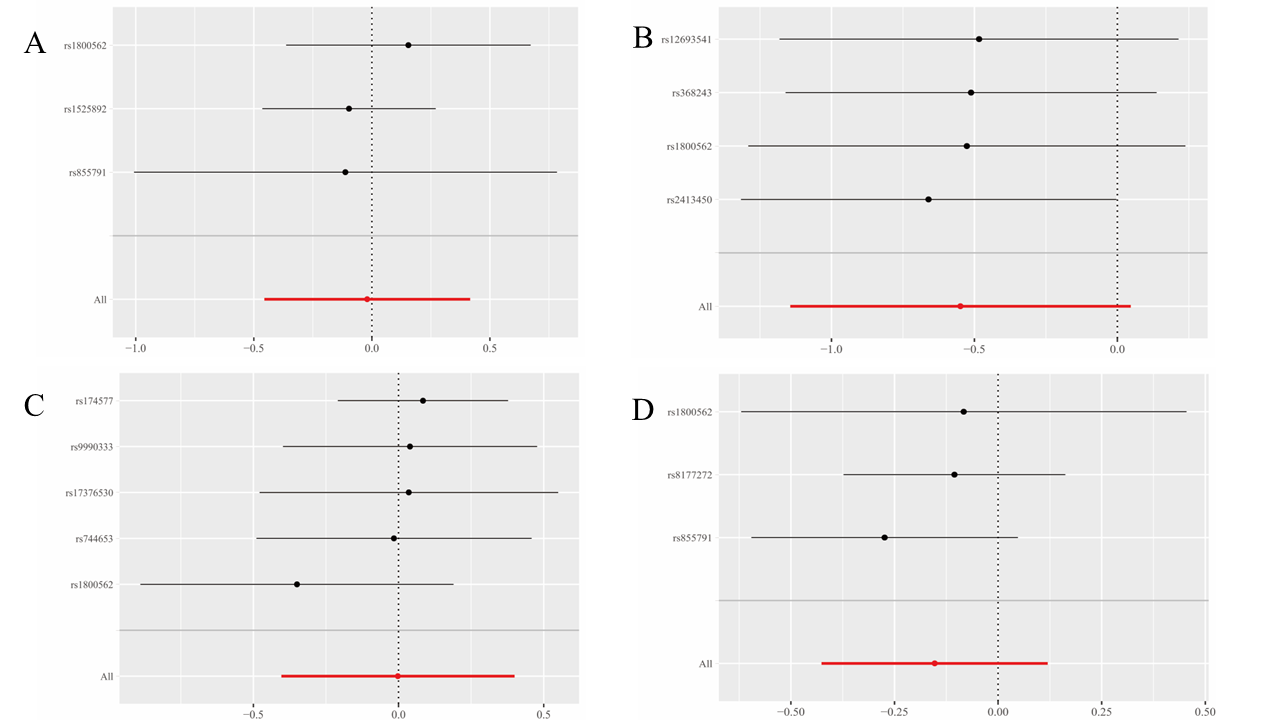
**

**Figure S3** The plots of the leave-one-out analysis. A Iron, B Ferritin, C transferrin, D Transferrin saturation.
